# Epidemiological disease burden and annual, nationwide health insurance treatment cost of female infertility based on real-world health insurance claims data in Hungary

**DOI:** 10.1186/s12913-025-12348-x

**Published:** 2025-03-04

**Authors:** Dalma Pónusz-Kovács, Tímea Csákvári, Luca Fanni Sántics-Kajos, Diána Elmer, Róbert Pónusz, Bettina Kovács, Ákos Várnagy, Kálmán Kovács, József Bódis, Imre Boncz

**Affiliations:** 1 Faculty of Health Sciences, Institute for Health Insurance, University of Pécs, 7621 Vörösmarty Utca 3, Pécs, Hungary; 2 National Laboratory On Human Reproduction, University of Pécs, 7624 Ifjúság Utca 20, Pécs, Hungary; 3 Department of Obstetrics and Gynecology, Medical School, Clinical Center, University of Pécs, 7624 Édesanyák Útja 17, Pécs, Hungary

**Keywords:** Female infertility, Cost, Prevalence, Epidemiology, Hungary

## Abstract

**Background:**

Infertility affects more than 50 million couples worldwide, resulting in a significant burden on individuals and society. Its prevalence ranges from 8–12% among developed countries. The growing number of patients poses an increasing challenge to the healthcare system and its funding. Our quantitative, descriptive, and cross-sectional study aimed to analyze the prevalence and annual nationwide health insurance treatment cost of female infertility in Hungary in 2019.

**Methods:**

We used claims data obtained from the Hungarian National Health Insurance Fund Administration (NHFIA). The number of patients, total and age-specific prevalence, annual health insurance expenditure, and the distribution of costs by age group were evaluated. Infertility was determined according to the World Health Organization International Classification of Diseases codes (N.97.0, N97.1, N97.2, N97.3, N97.4, N97.8, and N97.9) and the utilization of each healthcare service type. During the study descriptive statistics, correlation analysis and t-test were used.

**Results:**

In 2019, the NHIFA spent a total of 7.2 billion HUF (22.2 million EUR) on female infertility treatment in Hungary (33,151 women in outpatient care). The most significant costs were related to inpatient care (4.1 billion HUF, 12.7 million EUR). The highest number of patients and prevalence (650.4 per 100,000 women) were found in outpatient care. In inpatient care, the prevalence is substantially lower (206.7 per 100,000 women). Regardless of its type, female infertility mainly affects patients in the 30–39 years age group (number of patients: 18,156 women). The average annual health insurance expenditure per capita was 1,083 EUR.

**Conclusions:**

Reproductive health education, prevention, and medical screening play inevitable roles in the early stages of reproductive life to reduce the risk of infertility and decrease treatment costs.

## Background

In the 1960s, the beginning of a change in maternal preferences for having children was identified [1]. As a result, the mean age of a mother at the birth of her first child is now over 30 years in many Western countries [2, 3]. In this regard, Hungarians must face a similar tendency, as the mean maternal age at the birth of the first child is more than 29 years. The tendency of the ever increasing maternal age could be captured since almost the last four decades in Hungary. While in 1990 it was 22.9 years, in 2023 the mean maternal age was already 29,24 years [4]. As maternal age increases, there may be biological barriers that impact successful conception. Although it is not possible to quantify reproductive age precisely, fertility decline begins as early as between 25 and 30 years of age and becomes more significant after the age of 35 [5].

There are several approaches to the definition of infertility (medical, biomedical, psychosocial), [6] and the models often overlap. Infertility is defined by the World Health Organization (WHO) as a disease of the reproductive system in which clinical pregnancy does not occur after 12 months of regular and unprotected sexual intercourse [7, 8].

Infertility affects 48 million couples and 186 million people worldwide, 120 million of whom are women [9–11]. A total of 10–15% of reproductive couples struggle with unwanted childlessness [12]. The prevalence ranges widely, with a lower incidence in developed countries and higher rates in developing countries [13]. Developing countries often have limited statistical data and fewer healthcare-related resources, which might have a significant impact on the accessibility of screening and infertility treatment [14].

The number of children born in Hungary has been on a downward trend since the 1980s, with the effect that the number of live births has fallen by more than 38%. Since the social and political changes in Eastern Europe (1989), there has been an increase since the 1990’s in the mean age at childbearing, a postponement of adulthood, changes in family models and relationship patterns, and a decline in fertility levels. The combined effect contributes to the changing demographic conditions in Hungary. During the years of regime change, the mean maternal age with the first child was 21 years. Between 1993–1995 the mean maternal age decreased to 20 years, however from 1996 its increasing was detectable until 2004 when the mean maternal age was already 28–29 years. From 2004 onwards the mean maternal age has been 28–29 years, and it is still the same in nowadays [15, 16]. The fertility rate (FR) has been declining in Hungary since the 1990s (1990: 1.87) but has risen significantly in the last 10 years (2010: 1.25; 2021 = 1.59). Although both the live birth rate and the FR have increased in recent years, the country still faces negative demographic consequences. Most of these consequences come from the high level of natural decrease in the population, which exceeds the live birth rates by more than 40% every year [17]. The fertility rate in Hungary (1.53) is a little bit lower than the mean of the EU Member States (FR = 1.60), and in Slovenia (FR = 1.58), Germany (FR = 1.60), Czech Republic (FR = 1.63), Romania (FR = 1.64), Netherlands (FR = 1.66), Sweden (FR = 1.85), France (FR = 1.92), however it is higher than in Slovakia (FR = 1,48) and Poland (FR = 1.39) [18].

Infertility has a multifactorial origin: it may be exclusively female infertility (48.8%) or male infertility (10.1%), while in 26.6%, it may be due to a common cause or of unknown origin (14.4%) [19, 20]. However, in everyday life, the perception of infertility is often associated with female sex as the cause of the burden of failure to conceive, which may be due to several organ and hormonal abnormalities [21].

Assisted reproductive technologies (ARTs) are currently a breakthrough in the treatment of infertility, with a steady increase in the number of interventions performed by obstetrics and gynecology [22]. According to the European Society for Human Reproduction and Embryology (ESHRE), the number of healthcare institutions performing ART increased by more than 250% between 1997 and 2015 [23]. Clinical trials in the field of domestic ART (primarily in vitro fertilization, IVF) have shown significant results in terms of IVF efficacy [24–27].

Unintended childlessness is a major problem at both the individual and societal levels [28]. It can also place an increased burden on women and couples, as well as on healthcare and health insurance systems, especially in countries where the range of publicly funded services is currently limited [29, 30]. Therefore, infertility might also be seen as an ongoing and persistent health policy challenge [31, 32].

### The healthcare system in Hungary

The Hungarian healthcare system is highly centralized, and the involvement of the state is crucial in terms of resource generation, distribution, and also in the service provision. There is only one legally authorized entity which responsible for the fulfillment of domestic health insurance obligations. The health insurance system follows the Bismarckian tradition, (i.e., a social security contribution is the basis for access to publicly funded services, for which citizens are obliged to subscribe for) The economically active citizens (working-age population) have a legal responsibility to pay social security contributions, although contributions for full-time students and pensioners or other vulnerable individuals are covered by the central budget. Social security contributions cover 2/3 of public health expenditure; the remaining source is typically made up from governmental funds (taxes) and direct contributions (co-payments) from the population The market share of private health insurance interests is marginal, and it mostly covers the typical private healthcare services (i.e., dental, laboratory, medical imaging and other less cost-demanding examinations and interventions). However the utilization of private healthcare services can be accessible without private health insurance coverage, but in these cases the patients must face with direct obligation to pay for the caregiver [33–36].

The aim of the study was to determine the prevalence of female The aim of the study was to determine the prevalence of female infertility based on the data of the entire Hungarian publicly funded health care system independently from the type of the caregiver institution, furthermore, to analyzed the annual health insurance expenditure adhered to the infertility in 2019.

## Methods

The study database was based on the nationwide health insurance intel of the sole, publicly funded health insurance organization of Hungary. The National Health Insurance Fund Administration (NHIFA) is responsible for domestic health insurance purposes in Hungary. The study database was derived specifically from the public database of the National Directorate General for Hospitals of Hungary. The input for this database is provided by the NHFIA, as it contains real-world claims data for all Hungarian, publicly funded healthcare providers. This nationwide database, covering the whole Hungarian population and all the publicly financed health care providers allows us to perform unique analysis [37, 38].

Ethical approval was not provided for this study on human participants because the data were all accessed from the National Health Insurance Fund Administrations of Hungary. The analyzed was de-identified before access. The database was provided to our working group in accordance with the Declaration of Helsinki.

We identified female infertility with the N9.7 WHO International Classification of Diseases (ICD) codes (10th Revision). Inclusion criteria were the following codes: female infertility associated with anovulation (ICD N97.0), female infertility of tubal origin (ICD N97.1), female infertility with uterine origin (ICD N97.2), female infertility with cervical origin (ICD N97.3), female infertility associated with male partner factors (ICD N97.4), female infertility of another origin (ICD N97.8) and female infertility, unspecified (ICD N97.9) in caregiver institutions financed by NHIFA.

The analysis covered all publicly funded cases that were delivered by national healthcare providers. Thus, the database covers general practitioner, home care, nursing, patient transport, ambulance, laboratory care, medical imaging [(computer tomography (CT); magnetic resonance imaging (MRI); positron emission tomography (PET)], inpatient and chronic inpatient care, medical aids, pharmaceuticals, and medical equipment covered by itemized billing. Given that there were no data that supported the personal identification of patients, no ethical approval was needed for the delivery of the study.

To determine the number of patients with female infertility and the related health insurance costs of the treatment, diagnoses that appeared to be the main cause of care in inpatient and chronic inpatient care were also analyzed. The number of patients for whom ambulance services were used was included in the analysis, although PET diagnostics, disposable instruments, implantations, and medicaments falling under itemized billing were not utilized for infertility treatments during the analyzed year. For those treatments where the number of reported cases were n = 0 are not shown in the the graphical material and were not elaborated in text. The types of care concerned were PET, disposable instruments, implantations, and medicaments falling under itemized accounts and subsidized medical aids.

To determine the epidemiological characteristics of the diseases included in the study, the annual number of patients and the prevalence per 100,000 females by age group and type of care were evaluated. During the research period prevalence was delivered for the period between 1st January and 31st December 2019.For the analysis of the disease burden in the age groups, 7 groups were established: 0–19, 20–29, 30–39, 40–49, 50–59, 60–69, and over 70 years. The prevalence was analyzed and calculated from the type of treatment that had the highest number of patients (outpatient care). The prevalence was calculated from the type of care with the highest number of cases, which was outpatient care related to the female infertility. The number of female populations in Hungary was derived from the Hungarian Central Statistical Office (HCSO) database, which meant the basis for the evaluation [17].

The annual expenditure related to the utilization of the treatments was also evaluated from the perspective of the total study sample, the patients, and the type of treatment. The costs are expressed in euro (EUR), using the mean exchange rate of the Hungarian National Bank in 2019 (1 EUR = 325.35 HUF) [39].

For the data process the SPSS 27.0.1 (Statistics Pack-Age for Social Sciences 17.0) and Microsoft Office Excel 2016 were used. During the statistical assessment correlation analysis (Pearson-correlation), one-sample T-test and descriptive statistics were delivered. These statistical tests were delivered to determine the significant relationship between the variables (average age, average per capita health expenditure, number of cases per patient) and the degree of correlation. The values were analysed at 95% (*p* < 0.05) significance level. With the regression analysis the following evaluation was applied: if the correlation coefficient (r-value) is between 0.25–0.5 we considered it as a week correlation, if r-value is between 0.5–0.75 the correlation is moderately strong, while if the r-value is between 0.75–1.0 we considered it as a strong correlation.

The descriptive statistics were delivered to determine the mean age of patient, the sum and the proportion of the number of cases and the healthcare expenditre. If mean values were involved into the analysis, the confidence intervals (95%) was also set. For this assessment also a descriptive statistical method was delivered.

## Results

Table 1 shows the age-specific characteristics of the female population in Hungary and the incidence of female infertility in each age group. More than half of the patients (56.0%) were recorded in the 30–39 years age group, which was associated with the highest health care expenditure (53.8%), followed by the 40–49 years age group (26.8%) (Table 1).

**Table 1:** Number of patients in terms of age groups and diagnosis (WHO ICD) (NHIFA, HCSO, 2019).

Age groups	Total Hungarian female population 2019	Number of patients								Annual health insurance expenditure (EUR)	
		**N97.0 Anovulatio**	**N97.1 Tubal origin**	**N97.2 Uterine origin**	**N97.3 Cervical origin**	**N97.4 Male factor**	**N97.8 Other origin**	**N97.9 Unspecified**	**Total**		
**00–19**	928,293	39	5	2	1	7	15	280	349	10,395 €	0.00%
**20–29**	576,170	1,147	255	43	7	410	1,150	12,109	15,121	1,697,936 €	7.60%
**30–39**	632,698	2,865	1,248	149	39	2,278	4,991	40,350	51,920	11,941,403 €	53.80%
**40–49**	779,609	1,117	657	91	25	926	2,741	19,277	24,834	8,538,874 €	38.50%
**50–59**	627,392	32	25	2	2	2	30	291	384	13,449 €	0.10%
**60–69**	732,233	7	11	2	1	3	4	47	75	1,151 €	0.00%
**70 + **	820,540	9	1	2	0	1	7	28	48	736 €	0.00%
**Total**	**5,096,935**	**-**	**-**	**-**	**-**	**-**	**-**	**-**	**-**	**22,203,943 €**	**100.00%**

According to the type of treatment, the greatest number of patients were in outpatient care (*n* = 33,151). This was followed by pharmaceutical utilization (*n* = 21,624), laboratory care (*n* = 15,486), general practitioner care (*n* = 11,702), and inpatient care (*n* = 10,534).

The highest number of patients with “female infertility, unspecified” (ICD N97.9) was recorded. The ICD code N97.9 had the highest prevalence among general practitioners (8.

4.2%), outpatient care (78.9%), laboratory diagnostics (82.6%), and pharmaceutical utilization (73.6%). For the other ICD codes, a much lower number of patients were registered (Table 2).

**Table 2:** Annual number of patient according to different types of care of female infertility (NHIFA, 2019)

	**Annual number of patients**										
**Type of cares**	**N97.0**	**N97.1**	**N97.2**	**N97.3**	**N97.4**	**N97.8**	**N97.9**	**Total**	**Distribution**		
	**Anovulatio**	**Tubal origin**	**Uterine origin**	**Cervical origin**	**Male factors**	**Other origin**	**Unspecified**		**%**	**95%CI**	
**General practitioner care**	313	244	80	9	204	992	9,860	**11,702**	12.6%	12.0%	13.2%
**Patient transportation**	2	0	1	0	0	0	2	**5**	0.0%	0.0%	0.6%
**Ambulatory service**	0	0	1	0	0	0	5	**6**	0.0%	0.0%	0.7%
**Outpatient care**	1,952	483	64	6	706	3,780	26,160	**33,151**	35.8%	35.2%	36.3%
**Care in care centers**	1	0	0	0	0	6	16	**23**	0.0%	0.0%	0.7%
**Laboratory diagnostics**	833	95	22	2	119	1,618	12,797	**15,486**	16.7%	16.1%	17.3%
**CT, MRI**	3	0	1	0	0	10	59	**73**	0.1%	−0.6%	0.7%
**Outpatient care**	159	546	27	1	1,562	772	7,467	**10,534**	11.4%	11.3%	11.6%
**Chronic inpatient care**	1	16	0	0	1	0	85	**103**	0.1%	0.0%	0.8%
**Subsidized medicaments**	1,952	818	95	51	1,035	1,759	15,914	**21,624**	23.3%	22.8%	23.9%
**Subsidized medical aids**	0	0	0	0	0	1	1	**2**	0.0%	0.0%	0.6%
**Max level**	**1,952**	**818**	**95**	**51**	**1,562**	**3,780**	**26,160**	**33,151**	100.0%		

The number of patients according to age group was also analyzed. Among the population affected by infertility, women aged 30–39 years were the most affected (*n* = 18,156). Women aged 40–49 years (*n* = 7,882) and 20–29 years (*n* = 6,784) also represented a considerable annual number of cases. The remaining age groups had a negligible ratio from the whole study sample.

**Fig. 1:**
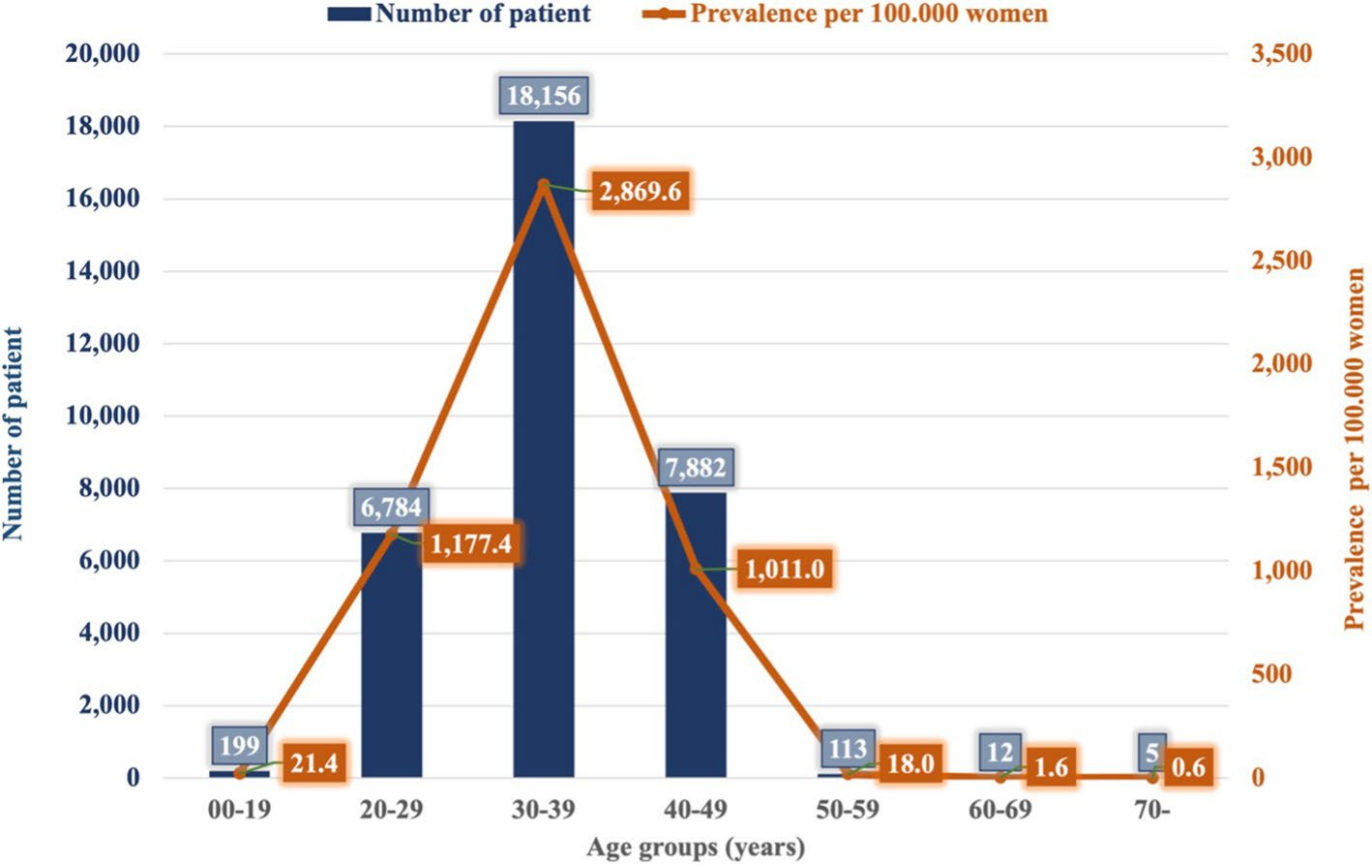
Number of patients and the prevalence of infertility cases

The prevalence was calculated based on outpatient care utilization, as it showed the highest occurrence. According to our results, the prevalence was greater among patients aged 30–39 years (2,869.6 cases/100,000 women) than among patients aged 20–29 years (1,177.4/100,000 women) or patients aged 40–49 years (1,011.0/100,000 women) Fig. 1.

**Fig. 2:**
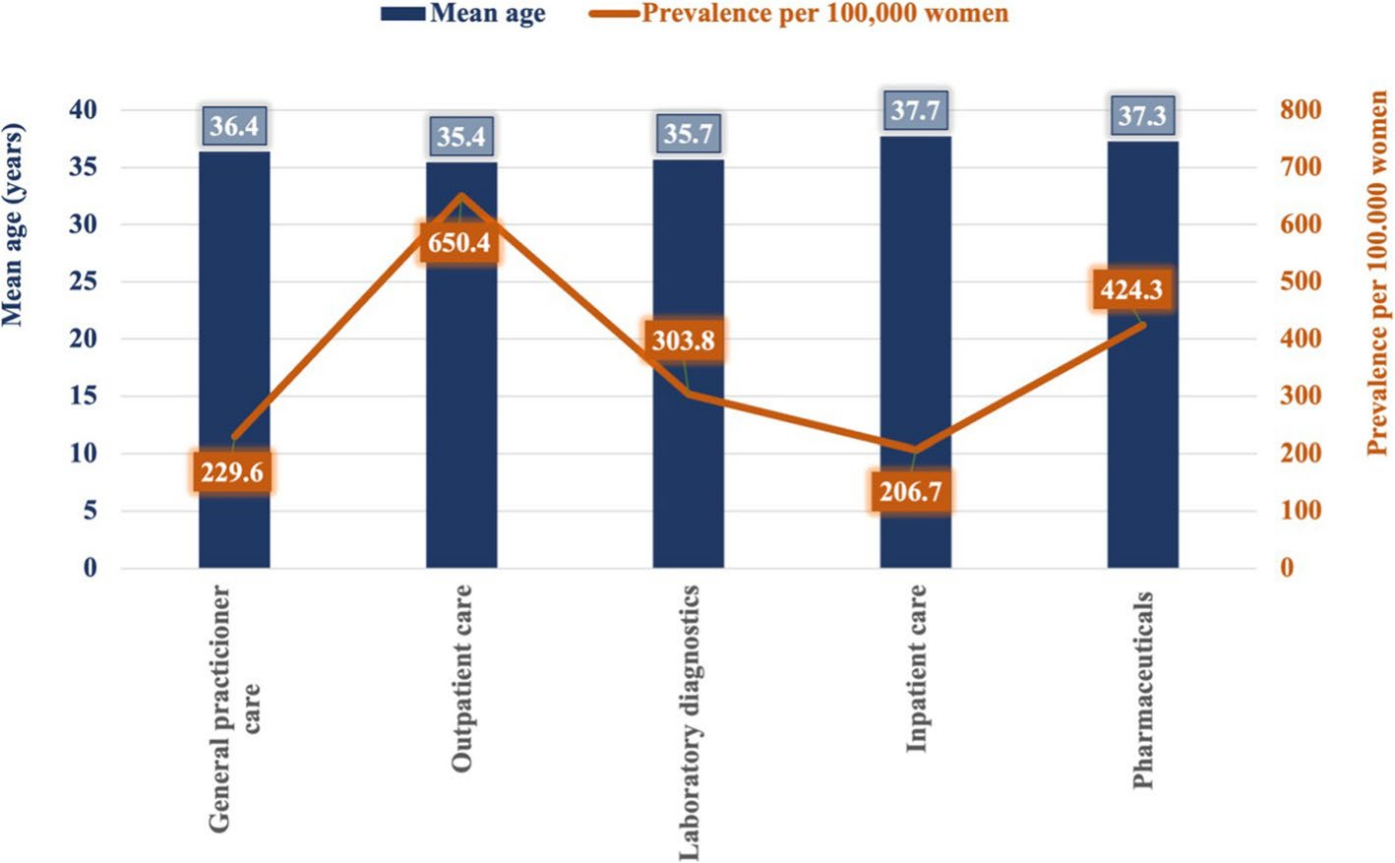
The mean age of infertility patients and its prevalence

The mean age of the infertility patients in the study sample was over 37 years [mean age = 37.3; 95% CI = 33.6–49.9]. The highest mean age was found among patients in inpatient care [37.71 years; CI (95%) = 23.6–53.0)], while the lowest was in outpatient care [35.40 years; CI (95%) = 24.1–36.7]. The mean ages of the participants in the pharmaceutical utilization [37.33 years; 95% CI = 21.8–40.5], general practitioner [36.42 years; 95% CI = 23.0–52.5], and laboratory diagnostic [35.71 years; 95% CI = 22.2–48.3] groups were not markedly different. It is important to highlight that the number of patients was the most significant in outpatient care, while it represented the lowest mean age of patients Fig. 2.

In 2019, the NHIFA spent more than 7 billion HUF on the treatment of infertility (7,224,052,939 HUF, = 22.2 million EUR), which represented 0.39% of total health insurance expenditure in Hungary in 2019 (1,826 billion HUF = 5,613 million EUR). The most significant percentage (57.2%) of the annual expenditure was related to inpatient care (4.1 billion HUF = 12.7 million EUR). There was also a considerable ratio of pharmaceutical utilization (2.7 billion HUF = 8.3 million EUR) to health insurance costs. The costs of outpatient care (201.2 million HUF = 618.5 thousand EUR), laboratory diagnostics (88.8 million HUF = 272 thousand EUR), general practitioner care (70.8 million HUF = 217 thousand EUR), chronic inpatient care (24.4 million HUF = 75 thousand EUR) and medical imaging services such as CT and MRI (3.8 million HUF = 11 thousand EUR) represented much lower amounts of the annual health insurance expenditure.

In 2019, a total of 11,702 number of patients were recorded in the GP care. In this year, the GP care had a total budget of 399.1 million EUR (129.7 billion HUF), of which female infertility represented for a marginal portion (0.05%) total of 217.0 thousand EUR (70.8 million HUF).

The NHIFA recorded “female infertility, unspecified” (ICD N97.9) as the most cost-demanding diagnosis (5.1 billion HUF = 15.8 million EUR), representing 71.1% of the total costs from the examined infertility-related disorders. The second highest health insurance expenditure was for “female infertility associated with male partner factors” (ICD N97.4), with an expenditure of 952.1 million HUF (= 2.9 million EUR). The health insurance expenditure for the diagnosis of “female infertility of other origin” (registered as ICD N97.8) amounted to 749.4 million HUF (= 2.3 million EUR). Significantly lower amounts were spent on “female infertility of tubal origin” (ICD N97.1) (275.5 million HUF = 846 thousand EUR) and “female infertility associated with anovulation” (ICD N97.0) (100.8 million HUF = 309 thousand EUR) Table 3.

**Table 3:** Annual health insurance treatment cost of infertility according to the types of care (EUR) (NHIFA, 2019)

**Type of care**	**Annual health insurance expenditure (EUR)**										
									**Distribution**		
	**N97.0 Anovulatio (EUR)**	**N97.1 Tubal origin (EUR)**	**N97.2** **Uterine origin (EUR)**	**N97.3** **Cervical origin** **(EUR)**	**N97.4** **Male factor (EUR)**	**N97.8** **Other origin** **(EUR)**	**N97.9** **Unspecified** **(EUR)**	**Total**	**%**	95%CI	
**General practitioner care**	4,517	3,566	1,246	105	2,869	16,111	189,386	**217,800**	6.1%	6.03%	6.23%
**Patient transportation**	37	0	5	0	0	0	70	**112**	0.1%	0.00%	0.65%
**Outpatient care**	20,629	10,169	748	91	12,518	69,455	504,866	**618,476**	6.2%	6.19%	6.31%
**Care in care centers**	4	0	0	0	0	95	169	**268**	0.0%	0.00%	0.22%
**Laboratory diagnostics**	11,118	849	218	16	1,227	10,734	248,738	**272,900**	0.9%	0.82%	0.89%
**CT, MRI**	475	0	143	0	0	1,066	10,196	**11,881**	5.8%	5.38%	6.22%
**Inpatient care**	84,731	627,458	17,351	1,729	2,414,451	1,208,021	8,361,868	**12,715,609**	31.6%	31.61%	31.66%
**Chronic inpatient care**	49	12,821	0	0	852	0	61,344	**75,066**	1.0%	0.00%	1.03%
**Pharmaceuticals**	188,434	191,869	3,247	1,875	494,559	998,106	6,413,734	**8,291,825**	48.2%	48.16%	48.23%
**Subsidized medical aids**	0	0	0	0	0	0	8	**8**	0.0%	0.00%	1.36%
**Total**	**309,993**	**846,733**	**22,958**	**3,815**	**2,926,476**	**2,303,588**	**15,790,379**	**22,203,943**	100.0%		
**Distribution (%)**	**1.4%**	**3.8%**	**0.1%**	**0.0%**	**13.2%**	**10.4%**	**71.1%**	**100.0%**			
95%CI	1.36%	3.77%	0.06%	−0.02%	13.14%	10.33%	71.10%				
	1.44%	3.85%	0.14%	0.06%	13.22%	10.41%	71.14%				

**Fig. 3:**
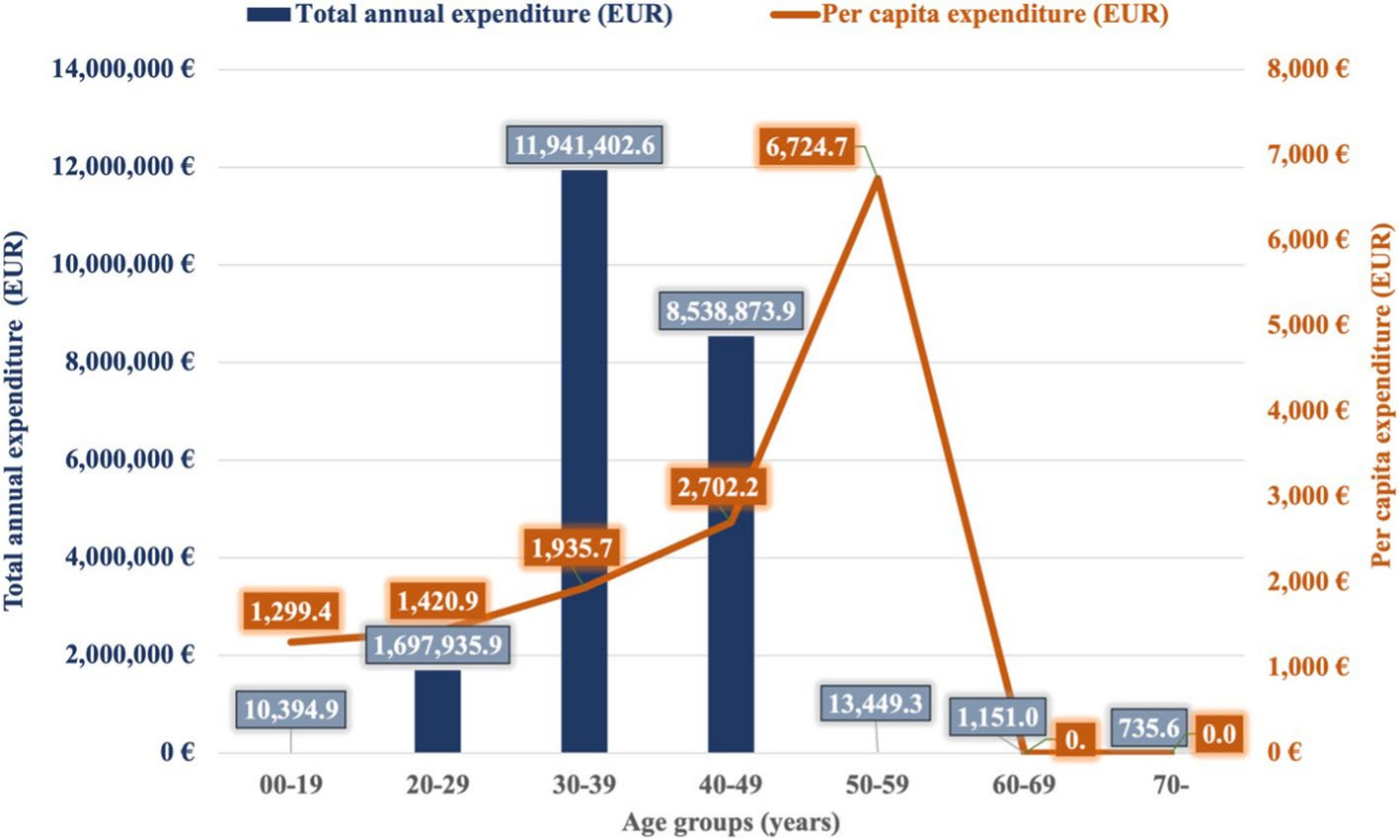
Total annual health insurance expenditures and average annual expenditures per capita by age group in 2019

According to the annual health insurance expenditures, the NHIFA spent the most public resources on the treatment of the 30–39 age group in the analyzed year (3.8 billion HUF = 11.9 million EUR). Remarkable health insurance costs were associated with treatment for the 40–49 age group (2.7 billion HUF = 8.53 million EUR), which was 38.5% of the total costs. The treatment of patients aged 20–29 years represented a significantly lower number of public resources (552.42 million HUF = 1.69 million EUR). According to the analysis of the per capita costs, the highest level of expenditure was reported for patients aged 50–59 years (2,187,869.8 HUF = 6,724.5 EUR), followed by those aged 40–49 years (879,152.7 HUF = 2,702.2 EUR) and those aged 30–39 years (629,783 HUF = 1,935.7 EUR) Fig. 3.

The average length of stay when female infertility was the main diagnosis in inpatient care was 4.05 days. The longest length of stay was recorded for patients with female infertility of another origin (ICD N97.8) (5.31 days), while the lowest was for female infertility with uterine origin (ICD N97.2) (2.37 days). There was no statistically significant correlation between the average length of stay and the mean age of the patients (*r* = −0.222, *p* = 0.295).

The average number of cases per patient was 2.38 in 2019, which was the highest for subsidized medicaments (6.22), outpatient care (3.50), and general practitioner care (3.02).

An inverse correlation was found between the annual number of cases per patient and the mean age of patients in outpatient care (*r* = −0.0514, *p* = 0.017): the number of cases per patient was higher among younger patients. A moderately strong correlation was captured in inpatient care: women with a higher mean age represented an increased number of cases (*r* = 0.695, *p* = 0.001). Our results revealed a significant association between the mean age of patients and the per capita costs of patient transportation (*r* = 0.585, *p* = 0.007) and subsidized medical aid (*r* = 0.702, *p* = 0.001).

Negative correlations were detected between the mean age of patients and the costs of therapies in general practitioner care (*r* = −0.072; *p* = 0.734), laboratory care (*r* = −0.404; *p* = 0.060) and inpatient care (*r* = −0.309; *p* = 0.148). Mostly patients with a lower mean age received inpatient treatment, which is the most cost-demanding type of medical service Table 4.

**Table 4:** Result of correlation analyses (source: own calculation based on data from the NHIFA, 2019)

Type of care	Mean age (years)	Health insurance expenditure per patient (EUR)	Annual number of cases per patient	Mean age vs. health insurance expenditure per patient		Mean age vs. annual number of cases per patient	
				**r value**	***p*** ** value**	***r*** ** value**	***p*** ** value**
**General practitioner care**	36.37	18.61	3.02	−0.072	0.734	−0.072	0.734
**Patient transportation**	87.66	22.38	2.00	0.585	0.007	-	-
**Ambulatory service**	31.79	0.00	1.00	-	-	-	-
**Outpatient care**	35.44	18.66	3.50	0.167	0.431	−0.514	0.017
**Care in care centres**	38.31	11.66	2.09	0.968	0.000	-	-
**Laboratory diagnostics**	35.70	17.62	2.33	−0.404	0.060	−0.483	0.025
**CT, MRI**	36.20	162.75	1.34	0.766	0.000	-	-
**Inpatient care**	37.73	1207.10	1.29	−0.309	0.148	0.695	0.001
**Chronic inpatient care**	37.79	728.79	1.00	0.359	0.094	-	-
**Subsidized medicaments**	37.27	383.45	6.22	0.288	0.178	0.143	0.497
**Subsidized medical aids**	44.80	4.20	2.50	0.702	0.001	-	-

## Discussion

Given the annual publicly funded utilization of infertility treatment in Hungary, the prevalence of female infertility was estimated to be 5.1%. The annual health insurance expenditure on certain disorders demanded 7.2 billion HUF (21.8 million EUR), which represented 0.39% of the total health insurance expenditure in 2019 in Hungary.

A total of 31,151 female patients diagnosed with infertility registered for outpatient care and received public health care, while 21,624 patients received medication. However, it is important to highlight that there might be an extensive difference between the number of patients affected by infertility and the number of patients who receive therapy. According to international studies, only 58% of infertility patients undergo infertility treatment [40–43]. In Hungary, there are no publicly available databases on private health service utilization, but it is important to note that the use of gynecological services is one of the highest among the utilization of private medical care [44].

Studies have reported an overall prevalence of infertility of 24.6% among women aged 20–40 years in a certain province of China [45], while in the UK, infertility is diagnosed in approximately 12.5% of women aged 16–74 years [46]. In Hungary, similar to Western European countries, more than 10% of couples of reproductive age face infertility problems [47]. International findings show that the number of patients affected by infertility is increasing annually [48].

In terms of the analyzed disorders, “female infertility, unspecified” (ICD N97.9) was the most common in the study population, which correlates with the results of other international studies [49, 50]. The experience of clinical practice confirms that these Hungarian claims data do not cover all gynecological diagnoses and report lower claims for the prevalence of infertility involving a specific female organ (N97.4, N97.3, N97.2, N97.1, N97.0) compared to previous studies on a similar topic [51]. This may be due to coding bias or a lower number of targeted medical imaging and laboratory tests (e.g., transvaginal ultrasound), which provide detailed information on the pelvic organs and could contribute significantly to an accurate diagnosis.

Patients often face the fact that the cause of infertility remains unknown [52]. Infertility increases steadily with age and is twice as likely to occur above the age of 35 years in those cases where the cause is still unknown [53]. Our study partially confirms this finding, as women aged 30–39 years are the most affected by this diagnosis in our sample, with a more than 2.5 times greater number of registered cases in this age group than in the 20–29 age group.

Pathology-induced utilization was most prevalent in outpatient care (*n* = 33,151; 37.8%). The following types of services were registered in outpatient care: gynecological examination, consultation, genetic counseling, and reproductive procedures. Almost a quarter of the patient population received drug treatment (*n* = 21,624; 23.3%), which was below the percentage reported in other nations [54]. Higher utilization rates were recorded for pharmaceutical utilization than for patient flow in inpatient care: 12.2 times for “female infertility associated with anovulation” (ICD N97.0) and 3.1 times for “female infertility of uterine origin” (ICD N97.2), while for “female infertility, unspecified” (ICD N97.9) and “infertility of other origins” (ICD N97.8) had 2.1 times higher utilization rates for pharmaceutical subsidies.

Among our study sample, only 16.7% of patients treated for infertility had a laboratory test. From this viewpoint, the Hungarian treatment pattern is underused, as other publications have reported a significantly higher rate of laboratory diagnostics [50].

The use of general practitioner consultations and treatments was rather low in our sample, with only 12.6% of the patients visiting their doctors (*n* = 11,702). General practitioners, who are the most familiar with patients' medical history, have an inevitable role in the timely initiation of patient investigations and referral to specialist clinics, as they have a key gatekeeper function in the provision of medical care [55, 56]. Furthermore, in contrast to international studies, no significant correlation could be detected between the number of patients and the mean age of patients for general practitioner care, but as with laboratory diagnostics, the number of cases per patient was greater in older patients than in younger patients [57].

The mean healthcare expenditure related to inpatient care and pharmaceutical utilization per patient is similar among Western European countries [58].

A significant part of the treatment cost was linked to inpatient care and the utilization of pharmaceuticals worldwide [59]. Hungary follows a similar pattern, with inpatient care and medicaments accounting for a significant share of expenditures on infertility. The market share of the annual expenditure related to inpatient care and pharmaceuticals was 94.6%, accounting for 21 million EUR on an annual basis.

ART interventions, which represent the most cost-demanding treatment for infertility, currently play a key role in the treatment of this disorder [60]. A survey among OECD countries showed that the cost of IVF procedures varies considerably between countries, which is closely linked to the specificities of their healthcare systems. In Hungary, the cost of IVF procedures is significantly lower than that in the United States (HUN is approximately 3,800 USD/cycle, while in the US, it is approximately 13,000 USD/cycle). The cost of ART in Hungary is 70% less than that in the US and 10–15% less than that in Western Europe [61]. The mean cost of inpatient care reimbursement for an IVF cycle in Hungary is 1,638 EUR/cycle, which is below the expenditure levels for interventions in the US and the UK [62].

In many Western societies, there is a growing pattern of postponing childbearing, with the proportion of women planning to have children after the age of 35 rising steadily. The trend also has a significant economic impact, with the increase in maternal age leading to a significant increase in healthcare expenditure [63]. In the early 2000s, more than 60% of the patients treated in infertility centers were women under 30 years of age, while today, the highest utilization of infertility treatment is among women aged between 30 and 49 years [64, 65].

Reproductive techniques are constantly evolving worldwide, while access to high-quality care is clearly improving. These components might even have the potential to create a false sense of security among members of the population in postponing the timing of childbearing [62, 66]. Nevertheless, it is also important to emphasize that age is one of the most significant determinants and might play a remarkable role in affecting the probability of both natural conception and successful pregnancy through reproductive procedures [67, 68]. The prevalence of infertility, the use of associated gynecological interventions and the number of cases per patient correlate with patient age [58].

Our results and international publications also highlight that the prevalence of infertility increases with age above 30 years [69]. In European countries, the prevalence of female infertility ranges from 6.6% to 16.7%. The prevalence of infertility in India is 8.9% among women of reproductive age and 13.1% in China [70, 71]. Our study revealed that Hungary is in the lower third among European countries in the prevalence of infertility.

The number of couples affected by infertility is increasing worldwide, making reproductive healthcare a necessity in an increasing number of countries. Reproductive healthcare can also be part of primary care, initially targeting the young population with a preventive approach. Prevention has particular importance in this regard to ensure patients’ awareness of regular screening and eliminate external factors that impair reproductive capacity before childbearing [72–74].

This study has several limitations. First, our research database did not include information on cases provided by private healthcare facilities. These data are not available publicly in Hungary. Given the lack of data, it was not possible to analyze the utilization and expenditures related to private healthcare, which may be of considerable interest to international audiences. If we had data from private healthcare, we would have assumed that the number of cases would have increased significantly.

Second, the study database did not contain information about the out-of-pocket expenditures of patients who received publicly funded infertility treatment. Third, the data did not include information about sick leave related to infertility, which has direct and indirect costs for both patients and society.

## Conclusions

The constant increase in the mean age of mothers at the birth of their first child is a common phenomenon observed across Europe. This challenge is compounded by the number of births that are delayed due to unwanted childlessness. A high prevalence of childlessness might be considered one of the most significant challenges in developed countries today. The epidemiological and health insurance relevance of female infertility in the publicly funded Hungarian health care system was described. Based on the Hungarian patient population, it can be concluded that the mean age of patients with infertility also shows an upward trend as the mean maternal age is extended. This leads to an increase in the cost of outpatient, inpatient, and pharmaceutical treatment. A greater cost of treatment per patient was registered for patients aged 40–49 years than for patients aged 30–39 years. It is important to highlight that maternal age might have a significant impact on the success rate of high-cost reproductive procedures. The increasing cost of treating infertility has become a significant burden on health insurance systems, society, and the domestic economy.

The current research shows that the utilization of primary care, laboratory, and medical imaging diagnostics is below international benchmarks. The responsibility of general practitioners in reproductive health promotion, starting at a younger age, primary and secondary prevention, regular laboratory tests, and early referral to specialist clinics should have particular importance in relieving the burden of infertility on affected couples.

It is important to highlight that appropriate patient education and early recognition of symptoms could reduce the direct and indirect costs of treatment for patients diagnosed with infertility, which would alleviate the burden of disease on patients and the funding pressures on the health insurance system in the country concerned.

## Data Availability

The data that support the findings of this study are available from the National Health Insurance Fund Administrations of Hungary, but restrictions apply to the availability of these data, which were used under license for the current study and are not publicly available. However, the data are available from the authors upon reasonable request and with the permission of the National Health Insurance Fund Administrations of Hungary but the statement is not present on the system.

## Abbreviations

ART: Assisted reproductive technology
CT: Computed tomography
ESHRE: European Society for Human Reproduction and Embryology
EU: European Union
EUR: Euro
HUF: Hungarian Forints
FR: Fertility Rate
HCSO: Hungarian Central Statistical Office
ICD: International Classification of Diseases
IVF: In vitro fertilization
LOS: Length of stay
MRI: Magnetic resonance imaging
NHIFA: National Health Insurance Fund Administration
PET: Positron emission tomography
WHO: World Health Organization
